# External Validation and Comparison of Current Scoring Systems in Encrusted Ureteral Stent Management: a Multicenter Study

**DOI:** 10.1590/S1677-5538.IBJU.2024.0500

**Published:** 2025-01-22

**Authors:** Mert Hamza Özbilen, Mehmet Çağlar Çakıcı, Erdem Kısa, Taylan Tığlı, Berk Yasin Ekenci, Burak Tüfekçi, Hilmi Sarı, İbrahim Güven Kartal, Ahmet Nihat Karakoyunlu, Gökhan Koç, Asıf Yıldırım, Hakan Erçil

**Affiliations:** 1 Health Sciences University Adana City Training and Research Hospital Department of Urology Adana Turkey Department of Urology, Health Sciences University Adana City Training and Research Hospital, Adana, Turkey; 2 Istanbul Medeniyet University Department of Urology Istanbul Turkey Department of Urology, Istanbul Medeniyet University, Istanbul, Turkey; 3 Medicana International Izmir Hospital Department of Urology Izmir Turkey Department of Urology, Medicana International Izmir Hospital, Izmir, Turkey; 4 Health Sciences University Izmir Tepecik Training and Research Hospital Department of Urology Izmir Turkey Department of Urology, Health Sciences University Izmir Tepecik Training and Research Hospital, Izmir, Turkey; 5 Ankara Etlik City Hospital Department of Urology Ankara Turkey Department of Urology, Ankara Etlik City Hospital, Ankara, Turkey; 6 Health Sciences University Kütahya Evliya Celebi Research and Training Hospital Department of Urology Kütahya Turkey Department of Urology, Health Sciences University Kütahya Evliya Celebi Research and Training Hospital, Kütahya, Turkey; 7 Izmir University of Economics Medicalpoint International Hospital Department of Urology Izmir Turkey Department of Urology, Izmir University of Economics Medicalpoint International Hospital, Izmir, Turkey

**Keywords:** Stents, Factor Analysis, Statistical, Urolithiasis

## Abstract

**Purpose:**

To compare the external validation of four existing scoring systems for encrusted ureteral stents (EUS) and their relationship with stent indwelling time, stone-free rates, multiple surgery sessions, multimodal procedures, and prolonged operation times exceeding 120 minutes in total.

**Materials and Methods:**

The data of 208 patients who underwent surgery for EUS reviewed. All EUSs were evaluated with 4 scoring systems: ESB (encrusted stone burden), FECal (forgotten, encrusted, calcified), KUB (kidney, ureter and bladder), V-GUES (visual grading for ureteral stone burden).

**Results:**

As the duration of stent indwelling time prolonged, a significant increase is observed in the scores of ESB, FECal, KUB and V-GUES systems (p<0.001). In multivariate logistic regression analysis, V-GUES score (p=0.025) and stent indwelling time (p=0.014) in stone-free rate, FECal grade (p<0.001) in multimodal procedure requirement, FECal (p=0.002) and V-GUES (p=0.032) scores in multiple surgery sessions, and stent indwelling time (p=0.019) and KUB score (p<0.001) in prolonged operation time were found to be predictors. When the area under receiver operating characterictic (ROC) curves (AUC) of the nomograms were examined, V-GUES score (AUC=0.685) in stone-free rate, FECal grade (AUC=0.780) in multimodal procedure requirement, FECal grade (AUC=0.845) in multiple surgery sessions, and KUB score (AUC=0.860) in prolonged operation time were found to be superior.

**Conclusions:**

The management of EUSs is often challenging for urologists. Although the current scoring systems for EUS differ somewhat, it is important to use scoring systems to guide the management of these patients.

## INTRODUCTION

Ureteral stents are widely used in urological practice to relieve upper urinary tract obstruction (1). Although ureteral stents are generally well tolerated by patients, they can cause pain, dysuria, bleeding, and lower urinary tract symptoms as a result of being a foreign body (2).

One of the complications that may occur due to a prolonged stent indwelling time is encrustation. Encrusted ureteral stent (EUS) is defined as a stent that cand not be removed using conventional cystoscopic methods and that requires additional intervention. EUS occurs in up to 13% of the cases (3). EUS can also cause renal failure and sepsis (4). Usually, ureteral stents are removed by cystoscopy without any problems. However, if there is encrustation additional procedures cand be required. Encrustation is the most challenging complication associated with ureteral stents, and requires a variety of complex procedures to manage (5).

Various scoring systems have been developed to enable accurate planning for EUS removal and ensure a stone-free status. The four scoring systems currently available are encrusted stone burden (ESB), forgotten, encrusted, calcified (FECal), kidney, ureter, and bladder (KUB), and visual grading for ureteral stone burden (V-GUES) (5–8) (supplementary appendix).

This study aimed to compare the external validation of four existing scoring systems for EUS and their relationship with stent indwelling time, stone-free rates, multiple surgery sessions, multimodal procedures, and prolonged operation times exceeding 120 minutes in total.

## MATERIALS AND METHODS

This clinical trial was approved by the Institutional Review Board (IRB No. 2022/04-15). The data of patients who underwent surgery for EUS between 2013 and 2023 in three tertiary care referral centers in three different regions of our country were retrospectively reviewed. Regardless of stent duration, patients whose stents were not encrusted or could easily be removed cystoscopically in a single attempt were excluded from the study. As a result of this review, data from 208 patients who met the inclusion criteria were evaluated.

The following data were collected: patient demographics (age, gender, diabetes mellitus (DM), Charlson comobidity index (CCI)), stent characteristics (symptom, indication of stent insertion, stent indweeling time, stone side, encrustation site, preoperative extracorporeal shock wave lithotripsy (ESWL), KUB score, FECal grade, V-GUES score, ESB, operative data (operation time, number of procedures, number of surgery sessions). The following outcomes were evaluated: stone-free rate, complications according to the Clavien–Dindo classification (9), hospitalization. Four available scoring systems for EUS were compared in relation to stent indwelling time, stone-free rates, multiple surgery sessions, multimodal procedures, and prolonged operation times exceeding 120 minutes in total.

Non-contrast computed tomography (NCCT) was performed in all patients after whose ureteral stents could not be removed cystoscopically in a single attempt to evaluate the surgery to be performed. The surgical method was decided according to the intraoperative EUS status. There are differences in brands and features of instruments and surgical equipment. Due to the large number of patients in the centers and the experience in complicated surgeries, ureterorenoscopy (URS), retrograde intrarenal surgery (RIRS), percutaneous nephrolithotomy (PCNL), ESWL could be applied without interruption in these centers. Multiple surgery sessions were defined as surgical interventions requiring more than one anesthesia, and multimodal procedures were defined as surgical interventions performed using more than one method. In patients who underwent multiple surgical sessions, the total operation time and hospital stay were reported. Patients were followed-up with NCCT one month after surgery. Success rate was defined as removal of the stent and stones <4 mm in NCCT performed one month after the procedure.

### Statistical Analysis

Data analysis was performed using Statistical Package for the Social Sciences version 22 for Windows (SPSS Inc., IBM, NY, USA). Pearson Chi-Square or Fisher's exact tests and the two-proportion Z test with adjusted p-values (Bonferroni method) were used for the comparison of independent categorical variables. The one-sample Kolmogorov-Smirnov test was applied to determine whether the data showed a normal distribution for the variables with quantitative values. One-way analysis of variance (ANOVA) was used for the variables of quantitative data that had a normal distribution after Tukey's post-hoc correction, and the Kruskal-Wallis test was used for the other variables. For comparison between the two groups, the t-test was used for the variables of quantitative data that had a normal distribution, and the Mann-Whitney test was performed for the other variables. Mean ± standard deviation was found in the data with normal distribution and median (minimum-maximum) values were recorded in the data without normal distribution. A receiver operating characteristic (ROC) curve was generated by plotting the sensitivity as a function of (1-specificity) to investigate the predictive values of the grading systems. Binary logistic regression analysis was performed to determine the independent risk factors for predicting the stone-free rate, multimodal procedure requirement, multiple surgery sessions requirements, and prolonged operation time. Multivariate analysis was performed using the significant parameters in the univariate analysis. Data were examined using 95% confidence intervals (CI). The likelihood of a type I error was considered α=0.05 for all tests.

## RESULTS

Considering the exclusion criteria, 208 patients who required surgical intervention due to EUS were included in the study. The demographic data and operative data of the patients are presented in Table-1 and Table-2. The mean age of the patients was 47.9 years. The indication for stent insertion was due to urinary system stone disease in 79.3%. The mean stent indwelling time was found to be 16.7 months. 46.2% of the encrustations occurred only in the bladder part of the ureteral stent. When the scoring systems were examined, the mean ESB, FECal, KUB and V-GUES scores were found to be 250 mm2, 2.2, 6.2 and 2, respectively. Although 47.1% of the patients underwent multimodal procedures and 16.8% underwent multiple surgical sessions, a stone-free status was achieved in 83.2% of the patients after all the interventions. Patients who were not stone-free were either lost to follow-up or had records that could not be accessed. Complications occurred at a rate of 17.8%, mostly Grade 1. Grade 4 complications developed in 2 patients: 1 patient with sepsis and 1 patient with septic shock. The mean hospital stay was 3.6 days.

**Table 1:** Demographic data of the patients.

Variables		
Number of patients		208
Mean age (years), mean ± SD		47.9 ± 15.0
**Gender, n (%)**		
	Female	72 (34.6)
	Male	136 (65.4)
Diabetes Mellitus, n (%)		36 (17.3)
CCI, median (min-max)		1 (0-7)
Symptomatic patients, n (%)		137 (65.9)
**Indication of stent insertion, n (%)**		
	URS	79 (38)
	RIRS-PCNL-ESWL	86 (41.3)
	Pyelolithotomy-Pyeloplasty-UNC	37 (17.8)
	Ureteral obstruction-Hydronephrosis-Acute renal failure	6 (2.9)
Stent indwelling time (months), (mean ± SD)		16.7 ± 18.5
≤ 6 mo, n (%)		74 (35.6)
6-12 mo, n (%)		38 (18.3)
13-24 mo, n (%)		50 (24)
>24 mo, n (%)		46 (22.1)
**Stone side, n (%)**		
	Right	109 (52.4)
	Left	90 (43.3)
	Bilateral	9 (4.3)
**Encrustation site, n (%)**		
	Kidney	23 (11.1)
	Ureter	17 (8.2)
	Bladder	96 (46.2)
	Kidney+Ureter	7 (3.4)
	Kidney+Bladder	23 (11.1)
	Ureter+Bladder	20 (9.6)
	Whole length	22 (10.6)

SD = standard deviation; CCI = charlson comorbidity index; URS = ureterorenoscopy; RIRS = retrograde intrarenal surgery; PCNL = percutaneous nephrolithotomy; ESWL = extracorporeal shock wave lithotripsy; UNC = ureteroneocystostomy; mo = month; n = number

**Table 2:** Operative data of the patients.

Variables		
Number of patients		208
Preoperative ESWL, n (%)		35 (16.8)
KUB score, (mean ± SD)		6.2 ± 2.9
**FECal grade, (mean ± SD)**		2.2 ±1.5
	Grade 1, n (%)	104 (50)
	Grade 2, n (%)	26 (12.5)
	Grade 3, n (%)	23 (11.1)
	Grade 4, n (%)	34 (16.3)
	Grade 5, n (%)	21 (10.1)
**V-GUES score, (mean ± SD)**		2.0 ± 1.1
	Type A, n (%)	96 (46.2)
	Type B, n (%)	37 (17.8)
	Type C, n (%)	47 (22.6)
	Type D, n (%)	28 (13.5)
Encrusted stone burden (mm2), mean ± SD		250.0 ± 491.4
Operation time (min), mean ± SD		74.0 ± 52.8
Operation time >90 min, n (%)		52 (25)
Operation time >120 min, n (%)		29 (13.9)
Operation time >180 min, n (%)		11 (5.3)
Number of procedures, mean ± SD		1.8 ± 1.1
Multimodal procedures, n (%)		98 (47.1)
Number of surgery sessions, mean ± SD		1.2 ± 0.5
Multiple surgery sessions, n (%)		35 (16.8)
Stone-free rate, n (%)		173 (83.2)
Complication, n (%)		37 (17.8)
**Clavien-Dindo classification, n (%)**		
	Grade 1	24 (11.5)
	Grade 2	8 (3.8)
	Grade 3A	3 (1.4)
	Grade 3B	0
	Grade 4A	1 (0.5)
	Grade 4B	1 (0.5)
	Grade 5	0
Hospitalization (day), mean ± SD		3.6 ± 4.7

SD = standard deviation; ESWL = extracorporeal shock wave lithotripsy; mo = month; n = number

The data obtained when grouped according to the stent indwelling time are shown in Table-3. When the relationship between stent indwelling time and operation times exceeding 90 minutes, 120 minutes and 180 minutes were investigated, no statistical difference was observed for operation times shorter than 120 minutes. Therefore, we evaluated prolonged surgery times exceeding 120 minutes in our study. At the same time, as the duration of the stent indwelling time was prolonged, a significant increase was observed in the ESB, FECal, KUB, and V-GUES systems (p<0.001).

**Table 3:** Relationship between stent indwelling time and scoring systems.

		≤ 6 mo (n=74)	7-12 mo (n=38)	13-24 mo (n=50)	>24 mo (n=46)	P value
Age, mean ± SD		47.6 ± 13.9	47.6 ± 15.1	50 ± 16.5	46.3 ± 15.0	0.666^A^
Gender						0.670
	Female	23 (31.1)	14 (36.8)	16 (32)	19 (41.3)	
	Male	51 (68.9)	24 (63.2)	34 (68)	27 (58.7)
Diabetes Mellitus, n (%)		13 (17.6)	3 (7.9)	13 (26)	7 (15.2)	0.162
Symptomatic patients, n (%)		48 (64.9)	24 (63.2)	33 (66)	32 (69.6)	0.932
**Stone side, n (%)**						0.125
	Right	38 (51.4)	25 (65.8)	22 (44)	24 (52.2)	
	Left	30 (40.5)	13 (34.2)	25 (50)	22 (47.8)
	Bilateral	6 (8.1)	0	3 (6)	0
Preoperative ESWL, n (%)		6 (8.1)	4 (10.5)	13 (26)	12 (26.1)	**0.012**^Z^
KUB score, mean ± SD		4.9 ± 2.0	5.5 ± 2.2	6.7 ± 2.7	8.4 ± 3.3	**<0.001**^A^
KUB score ≥ 9		3 (4.1)	3 (7.9)	11 (22)	20 (43.5)	**<0.001**^Z^
**FECal score**						
	Grade 1, n (%)	54 (73)	23 (60.5)	21 (42)	6 (13)	**<0.001**^K^
	Grade 2, n (%)	2 (2.7)	5 (13.2)	4 (8)	15 (32.6)	
	Grade 3, n (%)	11 (14.9)	2 (5.3)	3 (6)	7 (15.2)
	Grade 4, n (%)	5 (6.8)	7 (18.4)	15 (30)	7 (15.2)
	Grade 5, n (%)	2 (2.7)	1 (2.6)	7 (14)	11 (23.9)
FECal score ≥ Grade 3		18 (24.3)	10 (26.3)	25 (50)	25 (54.3)	**0.001**^Z^
**V-GUES Score**						**<0.001**^K^
	Type A, n (%)	45 (60.8)	22 (57.9)	18 (36)	11 (23.9)	
	Type B, n (%)	19 (25.7)	5 (13.2)	7 (14)	6 (13)
	Type C, n (%)	6 (8.1)	10 (26.3)	17 (34)	14 (30.4)
	Type D, n (%)	4 (5.4)	1 (2.6)	8 (16)	15 (32.6)
V-GUES Score ≥ C		10 (13.5)	11 (28.9)	25 (50)	29 (63)	**<0.001**^Z^
Encrusted stone burden (mm^2^), mean ± SD		89.6±116.9	310.5±603.6	214.6±528.2	496.6±613.4	**<0.001**^K^
Encrusted stone burden >250 mm^2^, n (%)		6 (8.1)	9 (23.7)	11 (22)	29 (63)	**<0.001**^Z^
Operation time >90 min, n (%)		26 (35.1)	6 (15.8)	9 (18)	11 (23.9)	**0.069**^Z^
Operation time >120 min, n (%)		1 (1.4)	3 (7.9)	7 (14)	18 (39.1)	**<0.001**^Z^
Operation time >180 min, n (%)		0a	1 (2.6)	3 (6)	7 (15.2)	**0.003**^Z^

A = One-Way ANOVA;

Z = Z Test;

K = Kruskal-Wallis test;

ESWL = extracorporeal shock wave lithotripsy; SD = standard deviation; mo = month; n = number

**Figure 1:**
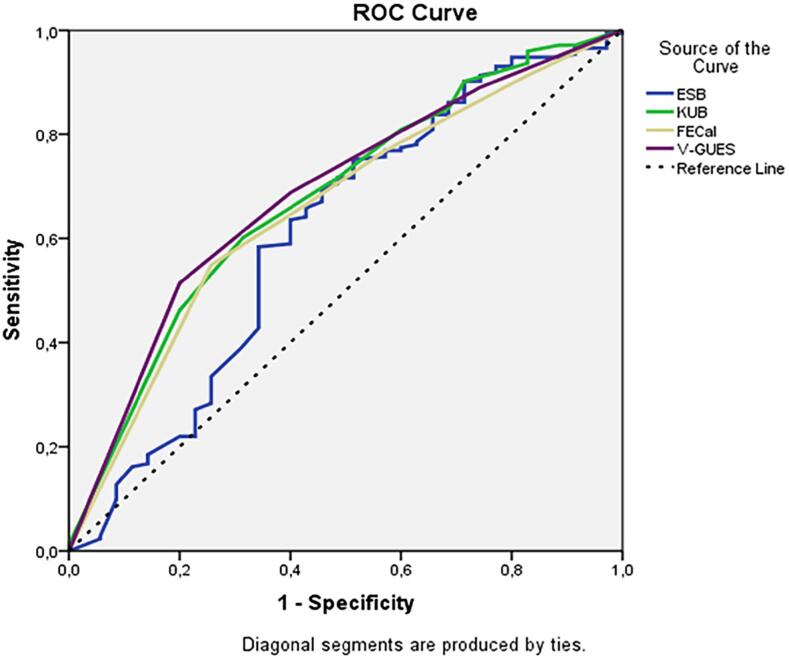
ROC curves of the nomograms for prediction of stone-free rate following surgery.

The multivariate logistic regression analysis of the factors predicting the postoperative stone-free rate is shown in Table-4. Accordingly, while a low V-GUES score (p=0.025) and short stent indwelling time (p=0.014) were associated with a high stone-free rate in the multivariate analysis, ESB, FECal grade and KUB score were not found to be predictive factors for the stone-free rate. In the ROC curve created using nomograms to predict the postoperative stone-free rate, the area under the curve (AUC) values were 0.610, 0.657, 0.677, and 0.685 for ESB, FECal, KUB and V-GUES, respectively (Figure-1).

**Table 4:** Multivariate logistic regression analysis of predicting factors for stone-free rate following surgery.

Binary Logistic Regression (n=208)						
	Univariate Model			Multivariate Model		
	OR	95% CI	P value	OR	95% CI	P value
Age	1.006	0.982-1.031	0.621	
Gender (Ref: female)	1.759	0.842-3.678	0.133
DM	1.310	0.471-3.645	0.605
Indications of stent insertion (Ref: URS)	0.855	0.550-1.328	0.485
Preoperative ESWL	0.511	0.215-1.214	0.128
Encrusted Stone Burden	0.999	0.999-1.000	0.063
KUB score	0.830	0.743-0.928	0.001
FECaL score	0.698	0.548-0.888	0.003
V-GUES score	0.553	0.397-0.770	<0.001	0.661	0.461-0.949	0.025
Stent indwelling time	0.968	0.950-0.985	<0.001	0.976	0.958-0.995	0.014

OR = odds ratio; CI = confidence interval; DM = diabetes mellitus; URS = ureterorenoscopy; ESWL = extracorporeal shock wave lithotripsy

**Figure 2:**
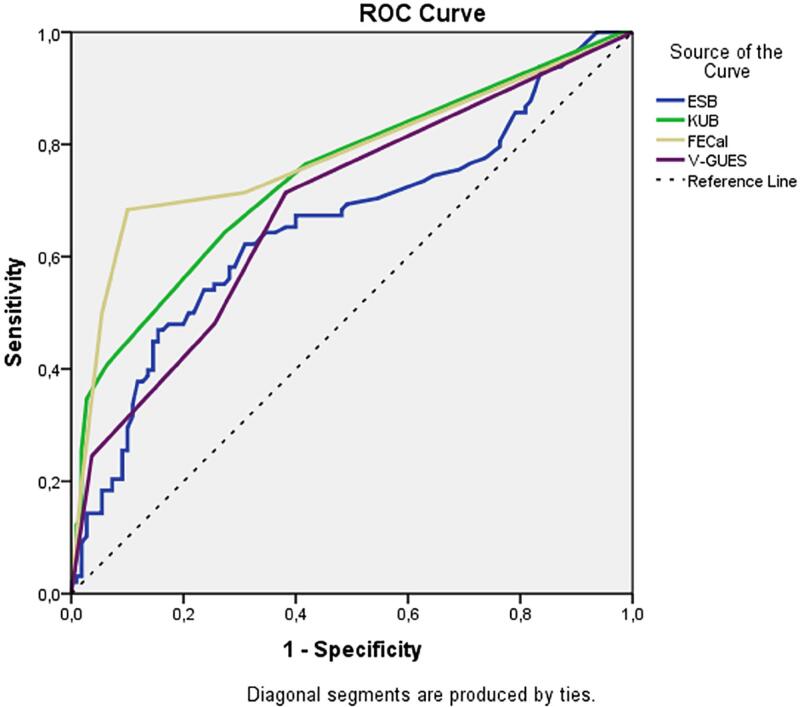
ROC curves of the nomograms for prediction of multimodal procedure requirement.

The results of the multivariate logistic regression analysis of the factors predicting the requirement for multimodal procedures are shown in Table-5. Accordingly, only an increase in the FECal grade was found to be a predictive factor for an increase in the number of multimodal procedures (p<0.001). In the multivariate analysis, ESB, KUB score, V-GUES score, and stent indwelling time were not associated with the requirement for multimodal procedures. The AUC values in the ROC curve created using the nomograms for the prediction of multimodal procedure requirements were 0.659, 0.780, 0.746, and 0.689 for ESB, FECal, KUB, and V-GUES, respectively (Figure-2).

**Table 5:** Multivariate logistic regression analysis of predicting factors for multimodal procedure requirement.

Binary Logistic Regression (n=208)						
	Univariate Model			Multivariate Model		
	OR	95% CI	P value	OR	95% CI	P value
Age	0.989	0.971-1.007	0.230			
Gender (Ref: female)	0.769	0.434-1.364	0.369			
CCI	0.899	0.755-1.069	0.229			
Indications of stent insertion (Ref: URS)	0.892	0.636-1.251	0.507			


Preoperative ESWL	1.870	0.892-3.919	0.097			
Encrusted Stone Burden	1.001	1.000-1.002	**0.006**			
KUB score	1.525	1.307-1.778	**<0.001**			
FECal score	2.587	1.983-3.373	**<0.001**	2.587	1.983-3.373	**<0.001**
V-GUES score	1.952	1.485-2.566	**<0.001**			
Stent indwelling time	1.020	1.003-1.037	**0.024**			

OR = odds ratio; CI = confidence interval; CCI = charlson comorbidity index; URS = ureterorenoscopy; ESWL = extracorporeal shock wave lithotripsy

**Figure 3:**
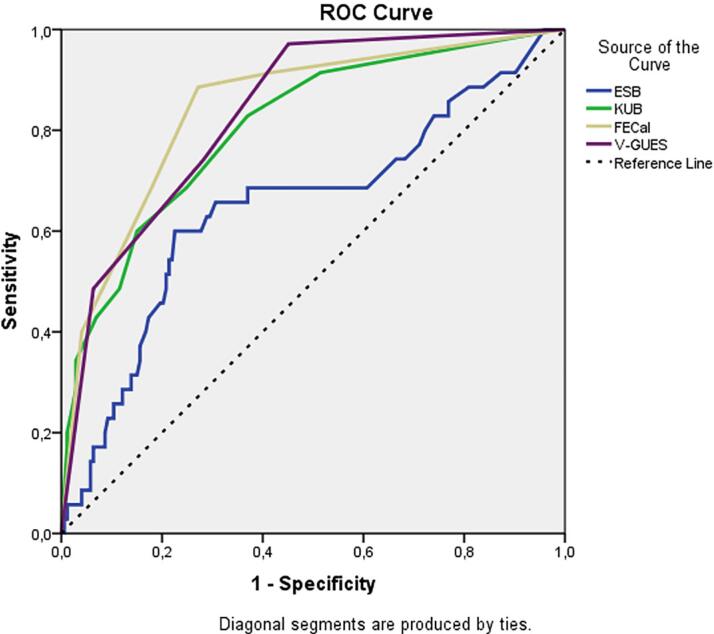
ROC curves of the nomograms for prediction of multiple surgery session requirement.

Multivariate logistic regression analysis of the factors predicting multiple surgery session requirements is shown in Table-6. Accordingly, it was found that an increase in FECal (p=0.002) and V-GUES (p=0.032) scores predicted an increase in the number of multiple surgical sessions. In the multivariate analysis, an increase in ESB, KUB score, and stent indwelling time did not predict the requirement for multiple surgical sessions. The AUC values in the ROC curve created using nomograms for the multiple surgery session requirements were 0.650, 0.845, 0.807, and 0.835 for ESB, FECal, KUB, and V-GUES, respectively (Figure-3).

**Table 6:** Multivariate logistic regression analysis of predicting factors for multiple surgery sessions requirement.

Binary Logistic Regression (n=208)						
	Univariate Model			Multivariate Model		
	OR	95% CI	P value	OR	95% CI	P value
Age	0.993	0.969-1.018	0.586			
Gender (Ref: female)	1.655	0.730-3.752	0.228			
CCI	0.854	0.665-1.097	0.217			
Indications of stent insertion (Ref: URS)	0.852	0.536-1.355	0.499			
Preoperative ESWL	0.846	0.485-1.812	0.317			
Encrusted Stone Burden	1.001	1.000-1.001	**0.041**		
KUB score	1.453	1.275-1.655	**<0.001**			
FECal score	2.729	1.971-3.780	**<0.001**	1.951	1.267-3.004	**0.002**
V-GUES score	3.565	2.313-5.492	**<0.001**	1.896	1.058-3.397	**0.032**
Stent indwelling time	1.015	0.998-1.032	0.075			

OR = odds ratio; CI = confidence interval; CCI = charlson comorbidity index; URS = ureterorenoscopy; ESWL = extracorporeal shock wave lithotripsy

**Figure 4:**
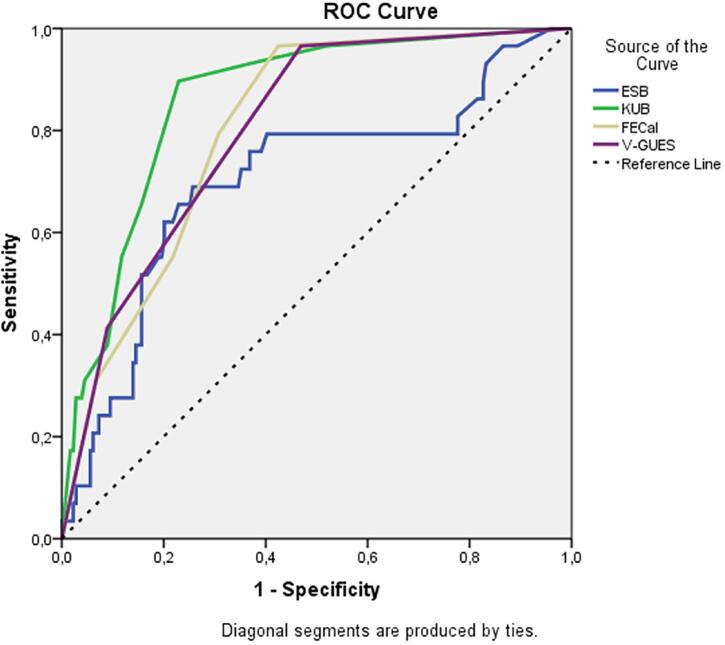
ROC curves of the nomograms for prediction of prolonged operation time (>120 min.).

Multivariate logistic regression analysis of the factors predicting the total duration of all surgical procedures ≥ 120 minutes is shown in Table-7. Accordingly, it was found that an increase in KUB score (p<0.001) and stent indwelling time (p=0.019) predicted prolonged operation time; however EBS, FECal grade, and V-GUES score were not predictive factors for operation time >120 minutes. The AUC values in the ROC curve created using the nomograms were 0.703, 0.804, 0.860, and 0.802 for ESB, FECal, KUB, and V-GUES, respectively, for operation times >120 minutes (Figure-4).

**Table 7:** Multivariate logistic regression analysis of predicting factors for operation time >120 minutes.

Binary Logistic Regression (n=208)						
	Univariate Model			Multivariate Model		
	OR	95% CI	P value	OR	95% CI	P value
Age	0.988	0.963-1.015	0.381			
Gender (Ref: female)	1.207	0.519-2.808	0.662			
CCI	0.953	0.739-1.228	0.709			
Indications of stent insertion (Ref: URS)	0.630	0.366-1.083	0.095			
Preoperative SWL	2.145	0.862-5.335	0.101			
Encrusted Stone Burden	1.001	1.000-1.001	**0.009**			
KUB score	1.470	1.284-1.683	**<0.001**	1.381	1.197-1.595	**<0.001**
FECaL score	2.102	1.560-2.831	**<0.001**			
V-GUES score	2.884	1.893-4.394	**<0.001**			
Stent indwelling time	1.048	1.025-1.070	**<0.001**	1.027	1.005-1.050	0.019

OR = odds ratio; CI = confidence interval; CCI = charlson comorbidity index; URS = ureterorenoscopy; ESWL = extracorporeal shock wave lithotripsy

In our study, preoperative ESWL was not found to be significant predictive factors for stone-free rates, multiple surgical sessions, multimodal procedures, and prolonged surgery times exceeding 120 minutes in multivariate analysis.

## DISCUSSION

Preoperative imaging is crucial to determine the treatment modality for EUS. Plain radiographs are insufficient to precisely determine the location and extent of calcification (10). Given the challenging nature of EUS management and the frequency of high-grade complications, NCCT is currently the preferred imaging method for evaluating the stone burden, degree of encrustation, and surrounding organs (11, 12). Preoperative and postoperative imaging of all patients in our study were performed using NCCT.

Surgery for patients with EUS carries high risk. There are many factors that put these patients at risk for postoperative complications, such as multiple comorbidities, large stone burden, and the presence of a potentially infected stent. Therefore, preoperative optimization is invaluable (13).

In a review conducted by Massella et al. on 1067 patients, it was found that more than half of the studies did not use any scoring system, making it difficult to draw a roadmap to guide the type and number of interventions required for stone-free status (1). After determining the degree of encrustation on EUS imaging, various grading systems are used to counsel patients and anticipate surgical difficulties that may arise during stent removal.

An ESB is a system that is calculated based on the encrustation volume. It is useful in determining the severity of encrustation, but it does consider the location of encrustation (5). FECal grade is an easy-to-apply scoring system. Although the treatment of encrustation at both ends of the stent was different, encrustation at the proximal or distal end did not affect the grade in this scoring. It was also developed in a study conducted on only 9 patients. However, it offers a treatment algorithm based on the grade (7). The KUB score evaluated encrustations involving the proximal and distal loops of the stent, separately. This is important in terms of the surgical method to be applied. However, this approach is complex and difficult to implement (14). The V-GUES score is the most up-to-date scoring system developed to help surgeons determine both the probability of EUS removal and the stone-free status. This is a visual scoring method based on NCCT and does not require calculations (8).

Although stent material, bacterial colonization, and patient-specific factors affect stent encrustation, the main risk factor for encrustation is stent indwelling time (15). Studies have shown that prolonged stent indwelling time is associated with a higher encrusted stone burden (p<0.001) (11, 16). The literature has mostly examined the relationship between stent indwelling time and the KUB and FECal scores. In all studies investigating the relationship between the total KUB score and stent indwelling time, prolongation of the stent indwelling time resulted in an increase in the KUB score (7, 14, 16, 17). In almost all studies that evaluated the FECal system, a positive relationship was found between increasing scores and stent indwelling time (6, 14, 17, 18). Only Lopes et al. found no association between stent indwelling time and FECal grade (p=011) (19). They attributed this result to their small sample size. Cicione et al. also stated that prolongation of stent indwelling time was associated with increased scores in the four existing scoring systems (20). Similarly, in our study, prolonged stent indwelling time caused an increase in all the scoring systems.

Achieving a stone-free status is one of the primary goals of EUS treatments. In the study by Lopes et al. no statistically significant relationship was found between the FECal grade and stone-free status (p=0.081) (19). Although one study reported that a total KUB score ≥ 9 was associated with a decreased stone-free rate, another study found that a total KUB score ≥ 9 was not associated with stone-free rates (7, 16). In a study comparing FECal and KUB scores by Guner et al. both scoring systems were found to be significant predictors of stone-free status in multivariate regression analysis (p<0.001) (17). Manzo et al. reported that the V-GUES score was associated with both stent removal and stone-free rates (8). In our study, the predictive factors for stone-free status in the multivariate analysis were V-GUES score (p=0.025) and stent indwelling time (p=0.014).

Multimodal procedures may be required to remove EUS and ensure stone-free status. There are conflicting results in the literature regarding multimodal procedures and scoring systems. In a study by Polat et al. an increase in ESB was found to be associated with multimodal intervention (p=0.012), while in another study, this relationship was not found (11, 18), Studies often report that a total KUB ≥ 9 is not associated with multimodal procedures, but an increase in FECal score correlates with the need for multimodal procedures (7,16,18). Guner et al. emphasized that the multimodal procedure was associated with the KUB score, but not with the FECal score (17). In a study by Saadi et al. FECal ≥ grade 3 was found to be a predictor of multimodal procedures. In this study, it was stated that both the KUB and FECal scores were useful for EUS removal, but the FECal score was advantageous in predicting the multimodal procedure (14). In our study, FECal was the only scoring system that predicted the requirement for multimodal procedures in multivariate logistic regression analysis (p<0.001).

In some series, it has been reported that stone-free status in patients with EUS can be achieved in a single surgical session; however, it has been shown that 1-3 surgical sessions are required in most cases (16). In a study by Weedin et al. stent indwelling time and ESB were not found to be significant in the multivariate analysis of factors predicting multiple surgery sessions (21). In contrast, Polat et al. found that an increase in ESB was associated with multiple surgical sessions (p=0.004) (11). Studies have reported that a total KUB score ≥ 9 is associated with multiple surgery sessions, and conversely, a total KUB score ≥ 9 is not associated with multiple surgery sessions (7, 16). In a study comparing FECal and KUB scores, total KUB ≥ 9 and FECal ≥ Grade 3 were found to be predictors of multiple surgery sessions in the multivariate regression analysis (14). In a study evaluating the V-GUES score, it was stated that multiple surgical sessions may be required as the score increases (8). In our study, the scoring systems that predicted the requirement for multiple surgery sessions in the multivariate logistic regression analysis were FECal (p=0.002) and V-GUES (p=0.032).

Prolonged operation time is a known risk factor for postoperative sepsis in stone surgery; therefore, attention should be paid to the operation time in surgeries performed for EUS (22). The scoring systems investigated in the literature for their relationship with prolonged operation time are KUB and FECal. When the FECal score was evaluated, both FECal≥ Grade 3 and high FECal grade were associated with prolonged operation time (14, 18, 19). Similarly, a total KUB score ≥ 9 and an increasing KUB score were also found to be associated with prolonged operation time (7, 14, 16). In our study, the KUB score (p<0.001) and stent indwelling time (p=0.019) were found to be significant in the multivariate analysis of the factors predicting the operation time exceeding 120 minutes.

The most important limitations of our study are that it was retrospective, the surgeries were performed by different surgeons with different endourological experiences, the stents were graded by different clinicians in different clinics, and the surgical equipment used was differed. The choice of surgery applied to encrusted stents was primarily based on urolithiasis guidelines, but while there are surgical recommendations according to stones in the guidelines, there is no clear recommendation for encrusted stents. In this respect, the decision-making process is left to the endourologist's preference. Other limitations of our study include the lack of data on the stent material and coating, and chemical analysis of the stones.

## CONCLUSIONS

EUSs are rare cases. Their management is a difficult procedure that requires experience and various equipment. In our study, among the scoring systems developed for EUS, V-GUES was found to be superior in predicting postoperative stone-free rate, FECal in predicting the need for multimodal procedures and multiple surgical sessions, and KUB in predicting prolonged surgical times exceeding 120 minutes. It may be useful to use all 4 KUB, FECal and V-GUES scoring systems to prepare both ourselves and the patient for the results of surgery in terms of different parameters with an individualized approach.

## ABBREVIATIONS

EUS: = Encrusted ureteral stents
ESB: = Encrusted stone burden
FECa l: = Forgotten, encrusted, calcified
KUB: = Kidney, ureter, bladder
V-GUES: = Visual grading for ureteral stone burden
CCI: = Charlson comorbidity index
DM: = Diabetes mellitus
ROC: = Receiver operating characteristic
AUC: = Area under curves
IRB: = Institutional review board
NCCT: = Noncontrast computed tomography

## APPENDIX:

### Supplementary Figure 1A Recurrence rate including only prospective trials.

*The ESB scoring system is based on the encrustation volume. It is expressed in square millimeters by multiplying the encrustation width by its length. An encrustation burden of >400 mm^2^ is considered severe (5). A 26-cm-long, completely encrusted stent with a 2-mm-wide encrustation corresponds to a stone burden of 520 mm^2^ (260 x2= 520 mm^2^)

*The FECal score is graded from 1 to 5 according to the location and size of the encrustation (6).

FECal ureteral stent grading system

- Grade 1 Minimal linear encrustations along either of the pigtail portions of the indwelling ureteral stent
- Grade 2 Circular encrustation completely encasing either of the pigtail portions of the indwelling ureteral stent
- Grade 3 Circular encrustation completely encasing either of the pigtail portions as well as linear encrustation of the ureteral aspects of the indwelling ureteral stent
- Grade 4 Circular encrustations completely encasing both of the pigtail portions of the indwelling ureteral stent
- Grade 5 Diffuse and bulky encrustations completely encasing both of the pigtail and ureteral portions of the indwelling ureteral stent

*The KUB scoring system is created by scoring the kidney (K), ureter (U) and bladder (B) sections of the EUS separately from 1 to 5 accorging to maximal diameter of encrustation and then summing them. Accordingly, a score between 3 and 15 is obtained (7).

KUB grading system

K—kidney

1. No evidence of calcification on the renal coil
2. Visible calcification on the renal coil, maximal width ≤ 5 mm, coil is not filled
3. Visible calcification on the renal coil, maximal width > 5 mm, coil is not filled
4. Coil is filled, calcification extends ≤ 5 mm outside of coil
5. Coil is filled, calcifications extends > 5 mm outside of coil (includes staghorn)

U—ureter

1. No evidence of calcification along the ureteral portion of stent
2. Single calcification along stent, maximal width ≤ 5 mm
3. Single calcification along stent, maximal width > 5 mm
4. Multiple calcifications along stent occupying < 50% of length of ureteral portion of stent, maximal width > 5 mm
5. Multiple calcifications along stent occupying ≥ 50% of length of ureteral portion of stent, maximal width > 5 mm

B—bladder

1. No evidence of calcification on the bladder coil
2. Visible calcification on the bladder coil, maximal width ≤ 5 mm, coil is not filled
3. Visible calcification on the bladder coil, maximal width > 5 mm, coil is not filled
4. Coil is filled, calcification extends ≤ 5 mm outside of coil
5. Coil is filled, calcifications extends > 5 mm outside of coil

*The V-GUES classification system is a visual system and varies from A to D, taking into account the severity and location of the encrustation (8).

V-GUES classification system
